# Identification and validation of ferroptosis-related gene signature in intervertebral disc degeneration

**DOI:** 10.3389/fendo.2023.1089796

**Published:** 2023-02-06

**Authors:** Qian Xiang, Yongzhao Zhao, Weishi Li

**Affiliations:** ^1^ Department of Orthopaedics, Peking University Third Hospital, Beijing, China; ^2^ Beijing Key Laboratory of Spinal Disease Research, Beijing, China; ^3^ Engineering Research Center of Bone and Joint Precision Medicine, Ministry of Education, Beijing, China

**Keywords:** lower back pain, intervertebral disc degeneration, nucleus pulposus, ferroptosis, oxidative stress

## Abstract

Lower back pain (LBP) is a leading cause of disability in the elderly and intervertebral disc degeneration (IDD) is the major contributor to LBP. Ferroptosis is a newly discovered programmed cell death, characterized by iron-dependent lethal lipid peroxidation. Growing evidence has shown that ferroptosis plays important roles in various human diseases. However, the underlying mechanism of ferroptosis in IDD remains elusive. This study is aimed to uncover the key roles of ferroptosis in the pathogenesis and progression of IDD comprehensively. To investigate the ferroptosis related differentially expressed genes (FRDEGs) in IDD, we analyzed the microarray data from the Gene Expression Omnibus (GEO) database. Then we performed functional enrichment analysis and protein-protein interaction (PPI) network analysis, and screened out the hub FRDEGs. To further evaluate the predictive value of these hub FRDEGs, we performed ROC analysis based on the GSE124272 dataset. A total of 80 FRDEGs were identified, including 20 downregulated and 60 upregulated FRDEGs. The FRDEGs were primarily involved in the biological processes of response to chemical, and response to stress. KEGG pathway enrichment analysis showed that the FRDEGs were mainly involved in ferroptosis, TNF signaling pathway, HIF-1 signaling pathway, NOD-like receptor signaling pathway, and IL-17 signaling pathway. Ten hub OSRDEGs were obtained according to the PPI analysis, including *HMOX1, KEAP1, MAPK1, HSPA5, TXNRD1, IL6, PPARA, JUN, HIF1A, DUSP1*. The ROC analysis and RT-qPCR validation results suggested that most of the hub FRDEGs might be potential signature genes for IDD. This study reveals that ferroptosis might provide promising strategy for the diagnosis and treatment of IDD.

## 1 Introduction

Lower back pain (LBP), a common musculoskeletal problem, is one of the leading causes of disability in the elderly worldwide (1, 2). It is acknowledged by a number of reports that the major contributor to LBP is intervertebral disc degeneration (IDD) (3, 4). Physiologically, the intervertebral disc (IVD) is an avascular structure of the human body in adults. The IVD is composed of three distinct regions, including the internal nucleus pulposus (NP), the peripheral annulus fibrosus (AF), and the inferior and superior cartilaginous endplates (CEP), with characterized cell types in each region respectively. Pathologically, the pathogenesis and progression of IDD is promoted by various and complicated factors, including aging, oxidative stress, inflammation, and mechanical stress, *etc.* The IDD process is characterized of degeneration of the NP, rapture of the AF, and calcification of the CEP (5, 6). However, the specific molecular mechanisms of disc degeneration remain elusive currently.

Ferroptosis is a newly discovered mode of regulated cell death, which differs from apoptosis, necrosis, and autophagy in morphological, biochemical, and genetic aspects (7). The cells that undergo ferroptosis have distinctive morphological characteristics, including cell membrane disruption and vesiculation, mitochondrial shrinkage with lessened cristae, mitochondrial membrane condensation and outer membrane rupture (8). In mechanism, ferroptosis is typified by intracellular iron-dependent lipid peroxidation and reactive oxygen species (ROS) accumulation to lethal levels (8, 9). Ferroptosis is implicated in various biological activities, including iron homeostasis, lipid peroxidation metabolism, glutathione (GSH) metabolism (10). Ferroptosis is negatively regulated by glutathione peroxidase 4 (GPX4), which is responsible for scavenging intracellular lipid peroxide through GSH (7, 11). It has been reported that the suppression of GPX4 or inhibition of GSH can effectively induce ferroptosis (11). Ferroptosis is also regulated by some other critical pathways, such as p53 signaling, Nrf2 signaling, Hippo signaling as well as mitochondrial signaling pathway (12).

Growing evidence has suggested that ferroptosis is interrelated with multiple pathophysiological contexts, including cancer, degenerative diseases, diabetes, cardiovascular diseases, *etc* (13, 14). Moreover, it has been demonstrated that ferroptosis might be also associated with some skeletal diseases, including osteoarthritis, osteoporosis, as well as rheumatoid arthritis (15). Osteoarthritis (OA) is a common degenerative joint disorder worldwide, and ferroptosis has been shown to play regulating roles in the pathogenesis and progression of OA. Recently, Miao et al. found that ferroptosis existed in OA, during which the key regulator GPX4 played critical roles in the chondrocyte cell death and extracellular matrix (ECM) degradation (16). Furthermore, by using single cell RNA sequencing analysis, Lv et al. (17) identified an important ferroptosis-associated target named TRPV1 in OA. And TRPV1 activation could protect chondrocytes from ferroptosis and mitigate the development of OA *via* modulating GPX4. A recent study revealed that some specific ferroptosis-related genes could be promising biomarkers for osteoporosis diagnosis and interventions, including the *ER*, *VDR*, *IL-6, COL1A1, COL1A2*, and *PTH* (18). Besides, growing evidence has also found the involvement of ferroptosis in the pathogenesis of rheumatoid arthritis, and targeting ferroptosis could be a promising therapeutic strategy for inflammatory arthritis.

Growing evidence has demonstrated that iron overload, closely associated with ferroptosis, is a common phenomenon in the aging process (19, 20). Unexpectedly, recent studies have revealed that this special type of cell death might also be related to IDD, a very common degenerative musculoskeletal disease that progresses with age. In 2021, Zhang et al. (21) established the IDD animal models and found that the levels of iron and Heme Oxygenase 1 (HO-1) were obviously elevated, and the levels of ferritin light chain markedly decreased in IDD compared to control. More recently, a study reported that iron overload could be an independent risk factor for IDD and it promoted endplate degeneration and calcification through oxidative stress and ferroptosis (22). However, the underlying mechanism of ferroptosis in IDD remains elusive and still needs further investigations. In the current research work, we aimed to explore the key roles of ferroptosis in the pathogenesis and progression of IDD comprehensively by using mature and recognized bioinformatic analysis methods, and hope to provide novel diagnostic and therapeutic targets for IDD.

## 2 Materials and methods

### 2.1 Data collection

The data used in this study is available in the Gene Expression Omnibus (GEO) repository (https://www.ncbi.nlm.nih.gov/geo/), with an accession number of GSE56081 for the differentially expressed genes (DGEs) identification dataset. The GEO dataset GSE124272 was used as a validation analysis dataset. The GSE56081 dataset contained five degenerated disc NP tissues and five control NP tissues (23). The GSE124272 dataset contained whole blood samples obtained from eight patients with IDD and eight healthy controls (24). The ferroptosis related genes, including ferroptosis markers, ferroptosis drivers, ferroptosis suppressors and unclassified genes, were acquired from the FerrDb online database (http://www.zhounan.org/ferrdb/current/). This study was approved by the Ethics Committee of Peking University Third Hospital.

### 2.2 Determination of DEGs and ferroptosis related DEGs

The DGEs in IDD were acquired by using the R package “limma”, and the criterions of identifying the DEGs were set as the fold change > 2 and the adjusted p value < 0.05. The FRDEGs were acquired by the intersection of DGEs based on GSE56081 and ferroptosis related genes based on FerrDb database by using the Venn diagram. Volcano plot of the DEGs, and hierarchical cluster heatmap of the FRDEGs were obtained by the R package “ggplot2”.

### 2.3 Functional enrichment analysis

The Gene Ontology (GO) analysis and Kyoto Encyclopedia of Genes and Genomes (KEGG) analysis were conducted for the FRDEGs and hub FRDEGs. The GO is an international standardized gene function classification system which is composed of three categories: biological process (BP), cell component (CC), as well as molecular function (MF). The GO analysis was conducted by the R package “clusterProfiler” (version 3.14.3) based on the GO annotations in R package “org.hs.eg.db” (version 3.1.0). The KEGG analysis was applied to determine related signaling pathways for FRDEGs. The KEGG analysis was conducted by the R package “clusterProfiler” (version 3.14.3) based on the latest KEGG pathway genes annotations, which were obtained from the KEGG rest API (https://www.kegg.jp/kegg/rest/keggapi.html). The corresponding item with a p value < 0.05 was considered statistically significant. The enriched items were plotted by using the R package “ggplot2”.

### 2.4 Protein-protein interaction network analysis

The PPI network analysis of the FRDEGs was conducted based on the STRING database v11.5 (https://cn.string-db.org/), a commonly used tool to evaluate the protein-protein interactions. The protein interaction pairs with score > 0.40 were further imported to the Cytoscape software v3.2 (https://cytoscape.org/) to establish PPI network. In the PPI network, the nodes represented the FRDEGs enriched in the STRING database, and the edges (connections between nodes) represented the interactions between different FRDEGs. The PPI score was obtained by using the degree analysis method in the CytoHubba plug-in, and the top ten significantly connected nodes were selected as the hub FRDEGs for further analysis.

### 2.5 Correlation analysis among the hub FRDEGs

To evaluate the relationships among these hub FRDEGs, the correlations among the hub FRDEGs were conducted by using Pearson correlation analysis. In the Pearson’s correlation analysis, the r value refers the correlation coefficient and was used to evaluate the effect size. And then the correlation matrix heatmap and the scatter plots were mapped by using the R package “ggplot2”.

### 2.6 ROC analysis of the hub FRDEGs

Receiver operating characteristics (ROC) analysis was applied to obtain the area under the curve (AUC) values by using the R software package “pROC” (version 1.17.0.1). In brief, the gene expression of the corresponding hub FRDEGs was obtained according to the GEO dataset GSE124272. Then the roc function of “pROC” was applied for ROC analysis, and the ci function of “pROC” was used to evaluate AUC and confidence interval. In the ROC curve, the sensitivity values were plotted on the Y-axis, and the false positive rates (1-specificity) values were plotted on the X-axis. The ROC curve with an AUC value >= 0.70 was considered to indicate an adequate predictive value.

### 2.7 Cell viability analysis

The rat NP cells were treated with different concentrations (0, 25, 50, 75 μM) of tert-butyl hydroperoxide (TBHP, Sigma-Aldrich, St. Louis, MO, USA) for 24 h to establish an *in vitro* IDD cell model, as reported previously (25, 26). The cell viability of the rat NP cells was evaluated by the cell counting kit- (CCK-) 8 assay (Dojindo, Japan). In brief, the cells were seeded in 24-well plates and incubated for 24 h, and then treated with TBHP with different concentrations. Subsequently, 20 μL of CCK-8 solution was added to each well with 200 μL culture medium. The cells were then incubated at 37°C for 2 h, and then the absorbance signal at 450 nm was detected using the SpectraMax iD3 spectrophotometer (Molecular Devices).

### 2.8 RNA extraction and RT-qPCR

Total RNA from the rat NP cells in each group was isolated by the SteadyPure Universal RNA Extraction Kit (AG21017, Accurate Biology, China) following the manufacturer’s instructions. RNA purity and concentration were determined by the DHS NanoPro 2020 spectrophotometer. Then the RNA was reverse-transcribed to cDNA by the Evo M-MLV Mix Kit with gDNA Clean for qPCR (AG11728, Accurate Biology, China). The qPCR assay was performed by using a SYBR Green Premix Pro Taq HS qPCR Kit (ROX Plus) (AG11718, Accurate Biology, China) with the QuantStudio 3 Real-Time PCR System (Applied Biosystems, USA). The qPCR conditions were set as follows: 95°C for 30 s, 40 cycles of 95°C for 5 s, and 60°C for 30 s; followed by a melt curve stage of 95°C for 15 s, 60°C for 1 min and 95°C for 15 s. Relative expression levels of genes was calculated by using the 2^−ΔΔCT^ method and normalized to GAPDH. The primers used in the present study are listed as follows (5’-3’). HMOX1 FORWARD: GGGTCAGGTGTCCAGGGAAGG; HMOX1 REVERSE: TGGGTTCTGCTTGTTTCGCTCTATC; KEAP1 FORWARD: TGCTCAACCGCTTGCTGTATGC; KEAP1 REVERSE: TCATCCGCCACTCATTCCTCTCC; MAPK1 FORWARD: TGAAGACACAGCACCTCAGCAATG; MAPK1 REVERSE: GGTGTTCAGCAGGAGGTTGGAAG; HSPA5 FORWARD: CGGAGGAGGAGGACAAGAAGGAG; HSPA5 REVERSE: ATACGACGGTGTGATGCGGTTG; TXNRD1 FORWARD: CACGGATGAGGAGCAGACCAATG; TXNRD1 REVERSE: CATACAGCCTCTGAGCCAGCAATC; IL6 FORWARD: ACTTCCAGCCAGTTGCCTTCTTG; IL6 REVERSE: TGGTCTGTTGTGGGTGGTATCCTC; PPARA FORWARD: ACGATGCTGTCCTCCTTGATGAAC; PPARA REVERSE: ATGATGTCGCAGAATGGCTTCCTC; JUN FORWARD: GGAAACGACCTTCTACGACGATGC; JUN REVERSE: GGAGGTGCGGCTTCAGATTGC; HIF1A FORWARD: CCGCCACCACCACTGATGAATC; HIF1A REVERSE: GTGAGTACCACTGTATGCTGATGCC; DUSP1 FORWARD: GCCACCATCTGCCTTGCTTACC; DUSP1 REVERSE: GATAATACTCCGCCTCTGCTTCACG; GAPDH FORWARD: GACATGCCGCCTGGAGAAAC; GAPDH REVERSE: AGCCCAGGATGCCCTTTAGT.

### 2.9 Statistical analysis

The data in IDD cell model were analyzed by GraphPad Prism software and were presented as means ± standard deviation of three independent experiments. The difference between groups was analyzed by unpaired Student’s t test. The correlation analysis among the hub FRDEGs was performed by Pearson analysis. A p value of less than 0.05 was considered to be statistically significant.

## 3 Results

### 3.1 Determination of the ferroptosis related differentially expressed genes

**Figure 1:**
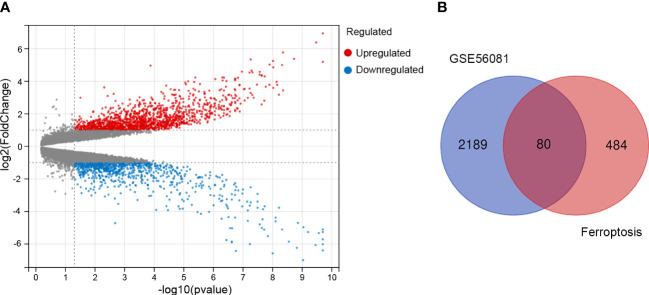
Identification of the differentially expressed genes between IDD and control groups. **(A)** The volcano plot of the differentially expressed genes in GSE56081 microarray data from the GEO database, in which the X-axis refers to the -log_10_(p value) and the Y-axis refers to the log_2_(fold change). The red dots represent the upregulated genes, and the blue dots represent the downregulated genes; the gray dots represent the genes without significant differential expression. **(B)** Venn diagram of the differentially expressed genes analyzed according to GSE56081 and the ferroptosis-related genes based on FerrDb database.

**Figure 2:**
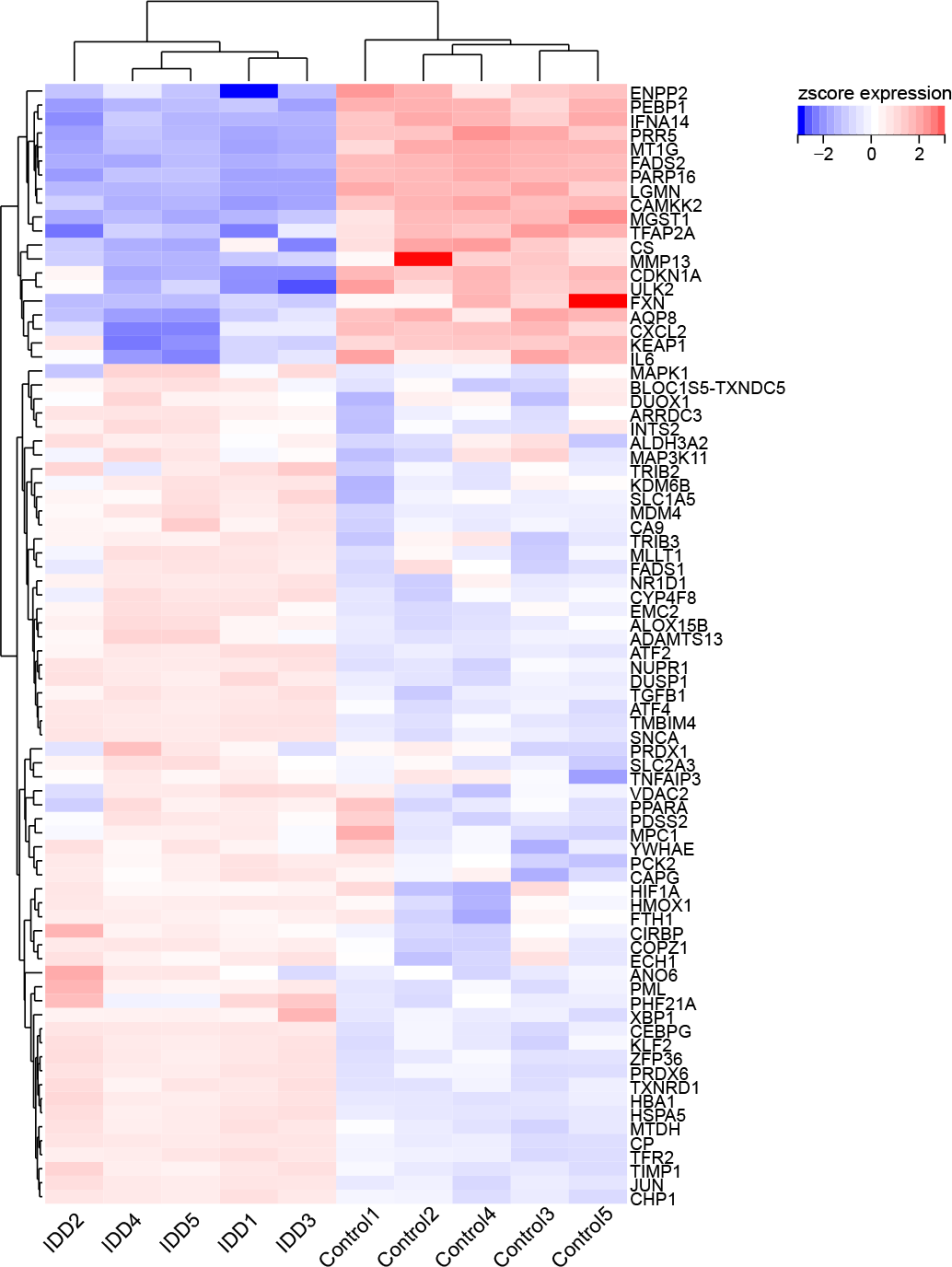
The hierarchical cluster heatmap of the 80 ferroptosis related differentially expressed genes (FRDEGs). The color scale indicates the relative gene expression of each sample. The red represents upregulated genes in IDD group compared to control, and the blue represents downregulated genes in IDD group. Among these FRDEGs, 20 FRDEGs are downregulated and 60 FRDEGs are upregulated.

First, we screened out the differentially expressed genes (DGEs) in IDD compared to control based on GSE56081 dataset. The volcano plot showed there are 2269 DGEs in total, including 847 downregulated and 1422 upregulated genes (**Figure 1A**). Subsequently, we intersected the DGEs in IDD with the ferroptosis-related genes based on FerrDb database. A total of 80 ferroptosis related differentially expressed genes (FRDEGs) were determined, as presented in the Venn diagram (**Figure 1B**). Among these 80 FRDEGs, 20 were downregulated and 60 were upregulated, as shown in **Table 1**. The hierarchical cluster heatmap demonstrated that the expression of ferroptosis-related genes in IDD group was obviously different from that in control group (**Figure 2**).

**Table 1:** A list of the 80 ferroptosis related differentially expressed genes (FRDEGs).

Gene symbols	Expression	Number
*FADS2, PARP16, LGMN, MT1G, CXCL2, CDKN1A, CAMKK2, IL6, AQP8, KEAP1, PEBP1, ULK2, MMP13, FXN, PRR5, IFNA14, ENPP2, MGST1, TFAP2A, CS*	Downregulated	20
*HBA1, TXNRD1, ZFP36, CEBPG, CP, KLF2, TFR2, ATF4, TMBIM4, TGFB1, INTS2, NUPR1, TIMP1, SLC2A3, JUN, TRIB3, PRDX6, ATF2, MPC1, SNCA, MTDH, ARRDC3, HSPA5, MDM4, MAPK1, HMOX1, ECH1, CIRBP, ALOX15B, PDSS2, CHP1, ADAMTS13, FADS1, NR1D1, BLOC1S5-TXNDC5, EMC2, PML, FTH1, CA9, TNFAIP3, CYP4F8, COPZ1, KDM6B, HIF1A, MAP3K11, MLLT1, ANO6, DUOX1, YWHAE, ALDH3A2, PPARA, PHF21A, XBP1, DUSP1, TRIB2, VDAC2, SLC1A5, PCK2, CAPG, PRDX1*	Upregulated	60

### 3.2 GO and KEGG function analysis of the FRDEGs

**Figure 3:**
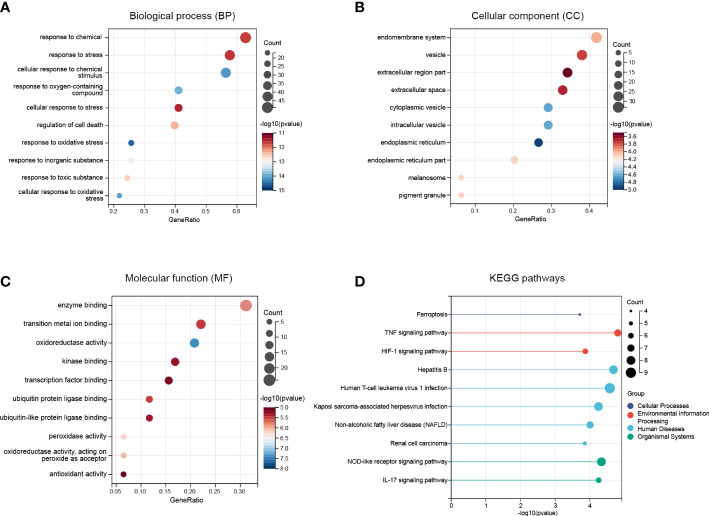
Functional enrichment analysis of the FRDEGs. **(A)** The top 10 significantly enriched GO terms in the category of biological process (BP) for the FRDEGs. **(B)** The top 10 significantly enriched GO terms in the category of cellular component (CC). **(C)** The top 10 significantly enriched GO terms in the category of molecular function (MF). **(D)** Kyoto Encyclopedia of Genes and Genomes (KEGG) pathway enrichment analysis for the FRDEGs.

The Gene Ontology (GO) is an international standardized gene function classification system which is commonly used to classify the predicted genes function. There are three main GO categories: biological process (BP), cellular component (CC), and molecular function (MF). As for the GO enrichment analysis for biological process, these FRDEGs are mainly involved in response to chemical, response to stress, and cellular response to chemical stimulus (**Figure 3A**). As shown in **Figure 3B**, the most significantly enriched GO items for cellular component are endomembrane system, vesicle, and extracellular region part. As for the molecular function, these FRDEGs are mainly involved in enzyme binding, transition metal ion binding, and oxidoreductase activity (**Figure 3C**). And then, we conducted the Kyoto Encyclopedia of Genes and Genomes (KEGG) analysis for the 80 FRDEGs. As indicated in **Figure 3D**, results show that the FRDEGs are primarily involved in the following significant pathways: Ferroptosis, TNF signaling pathway, HIF-1 signaling pathway, NOD-like receptor signaling pathway, and IL-17 signaling pathway, *etc.*

### 3.3 PPI analysis of the FRDEGs

**Figure 4:**
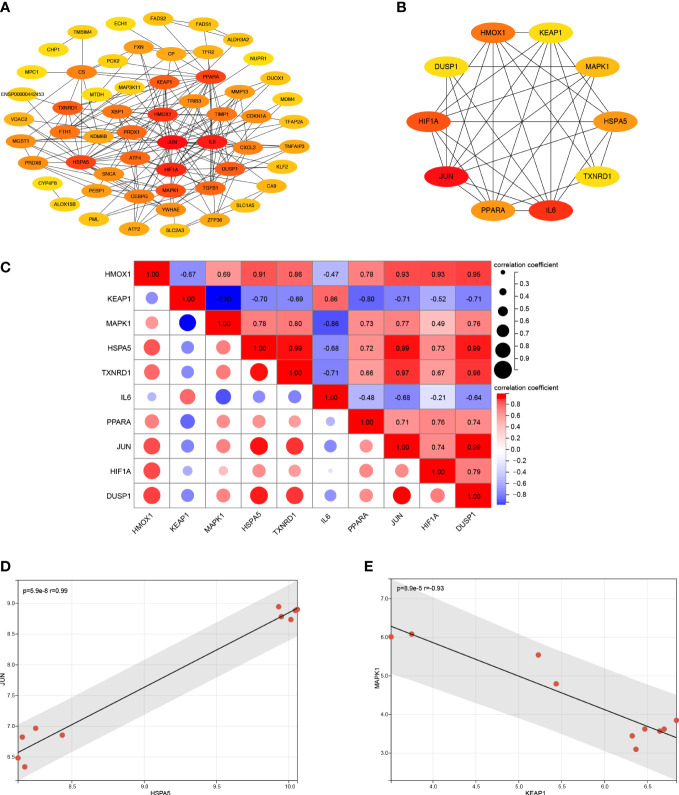
PPI analysis of the FRDEGs. **(A)** PPI analysis of these FRDEGs. **(B)** The top 10 hub FRDEGs. **(C)** Correlation analysis among the top 10 hub FRDEGs by Pearson analysis. **(D)** Correlation analysis between *HSPA5* and *JUN* by Pearson analysis. **(E)** Correlation analysis between *KEAP1* and *MAPK1* by Pearson analysis.

The PPI network analysis was performed based on the STRING database and visualized by Cytoscape software (**Figure 4A**). Then we determined the top ten hub FRDEGs based on the PPI score, including HMOX1, KEAP1, MAPK1, HSPA5, TXNRD1, IL6, PPARA, JUN, HIF1A, and DUSP1, as shown in **Figure 4B**. Detailed information of these hub FRDEGs was presented in **Table 2**. Among these hub FRDEGs, KEAP1 and IL6 were downregulated in IDD, and the rest six hub genes were obviously upregulated. Most of these hub genes had either promoting or inhibiting effects on ferroptosis, and the potential roles of TXNRD1 on ferroptosis was not fully understand. Next, we performed Pearson correlation analysis to evaluate the relationships among these hub FRDEGs (**Figure 4C**). The most negatively related pair was KEAP1-MAPK1, and the most positively related pairs were HSPA5-JUN, HSPA5-TXNRD1, HSPA5-DUSP1, and DUSP1-JUN. The correlation analysis of HSPA5-JUN pair was presented in **Figure 4D**, with a r value of 0.99 and p value < 0.01. The correlation analysis of KEAP1-MAPK1 pair was shown in **Figure 4E**, with a r value of -0.93 and p value < 0.01.

**Table 2:** Detailed information of the top 10 hub FRDEGs.

Gene symbols	Gene full names	Expression	Log2(fold change)	P value	Ferroptosis regulation
*HMOX1*	Heme oxygenase 1	Upregulated	1.60	<0.01	Driver, Suppressor
*KEAP1*	Kelch like ECH associated protein 1	Downregulated	-1.60	<0.01	Driver
*MAPK1*	Mitogen-activated protein kinase 1	Upregulated	1.69	<0.01	Driver
*HSPA5*	Heat shock protein family A (Hsp70) member 5	Upregulated	1.77	<0.01	Suppressor
*TXNRD1*	Thioredoxin reductase 1	Upregulated	3.56	<0.01	Unclassified
*IL6*	Interleukin 6	Downregulated	-1.64	<0.01	Driver, Suppressor
*PPARA*	Peroxisome proliferator activated receptor alpha	Upregulated	1.06	<0.01	Suppressor
*JUN*	Jun proto-oncogene	Upregulated	2.16	<0.01	Suppressor
*HIF1A*	Hypoxia inducible factor 1 subunit alpha	Upregulated	1.22	<0.01	Driver, Suppressor
*DUSP1*	Dual specificity phosphatase 1	Upregulated	1.04	<0.01	Unclassified

### 3.4 Function analysis of the hub FRDEGs

**Figure 5:**
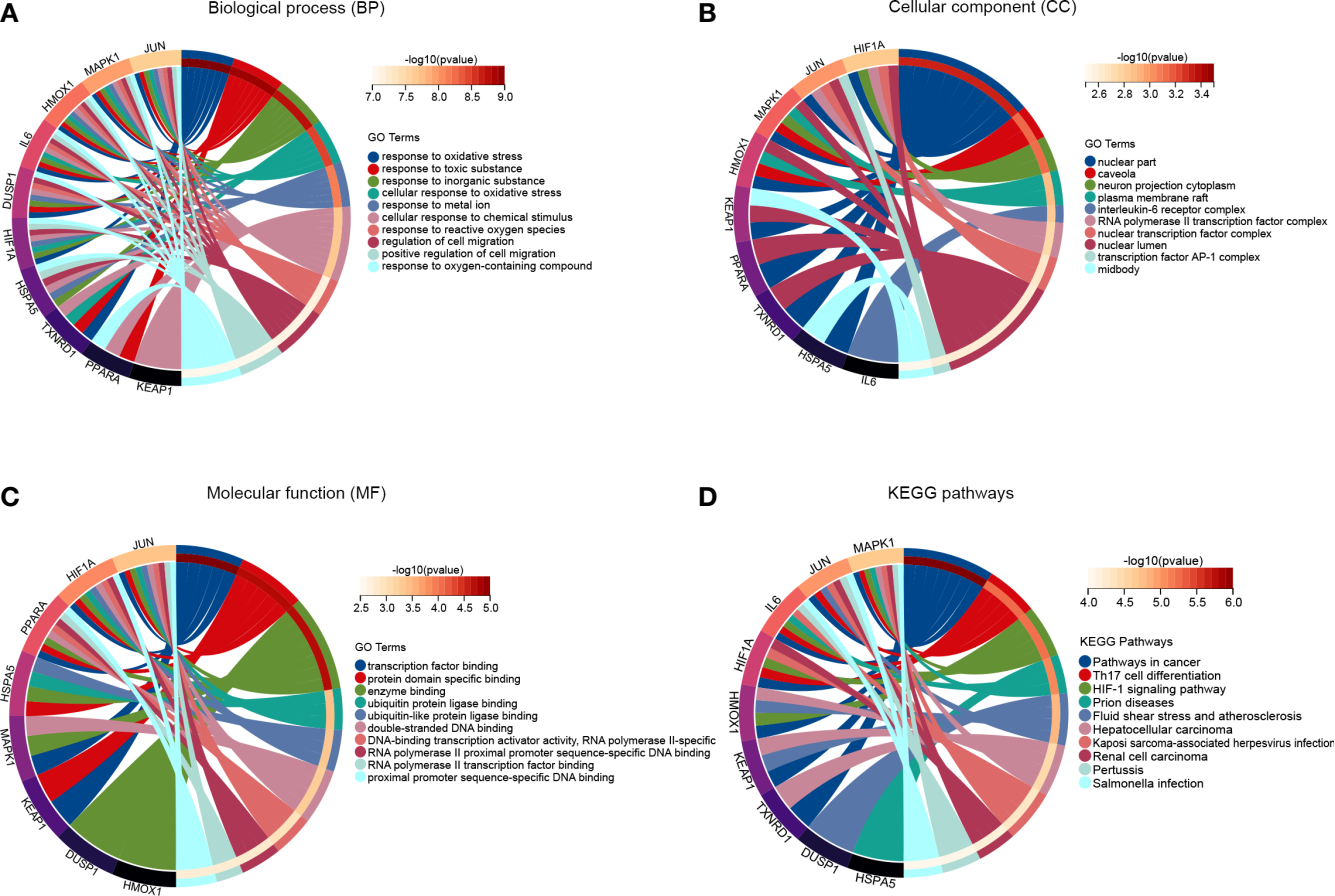
Function analysis of the hub FRDEGs. **(A)** The top 10 significantly enriched GO terms in the category of biological process for these hub FRDEGs. **(B)** The top 10 significantly enriched GO terms in the category of cellular component. **(C)** The top 10 significantly enriched GO terms in the category of molecular function. **(D)** KEGG pathway analysis for these hub FRDEGs.

To further investigate the potential molecular functions of the top ten hub FRDEGs, we performed functional and pathway enrichment analysis. As for the GO enrichment analysis in the category of biological process, these hub FRDEGs are mainly involved in response to oxidative stress, response to toxic substance, response to inorganic substance, and cellular response to oxidative stress (**Figure 5A**). In the category of cellular component, the most significantly enriched GO items include nuclear part, caveola, neuron projection cytoplasm, and plasma membrane raft (**Figure 5B**). In the molecular function, the FRDEGs are mainly associated with transcription factor binding, protein domain specific binding, enzyme binding, and ubiquitin protein ligase binding (**Figure 5C**). Subsequently, we performed KEGG pathway enrichment analysis for the ten hub genes. As shown in **Figure 5D**, it is suggested that the most meaningful and significantly enriched pathways include Th17 cell differentiation and HIF-1 signaling pathway.

### 3.5 The validation of hub FRDEGs in GSE124272 dataset

**Figure 6:**
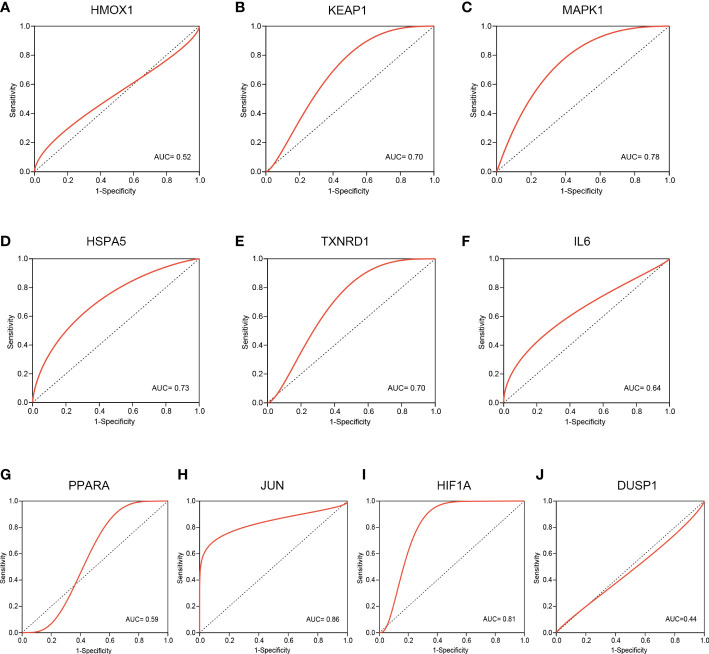
Validation of these hub FRDEGs based on the GSE124272 dataset. **(A–J)** ROC analysis of the ten hub FRDEGs, including *HMOX1, KEAP1, MAPK1, HSPA5, TXNRD1, IL6, PPARA, JUN, HIF1A*, and *DUSP1*. The X-axis represents the (1-Specificity), and the Y-axis represents the Sensitivity.

To evaluate the predictive value of these hub FRDEGs, we performed ROC analysis based on the GSE124272 dataset. The ROC curve reveals that the AUC values of six hub genes are greater than or equal to 0.70, including *KEAP1, MAPK1, HSPA5, TXNRD1, JUN*, and *HIF1A* (**Figures 6A–J**). The above data suggested that these six hub FRDEGs have potential predictive values for IDD.

### 3.6 Expression validation of hub FRDEGs in IDD cell model

**Figure 7:**
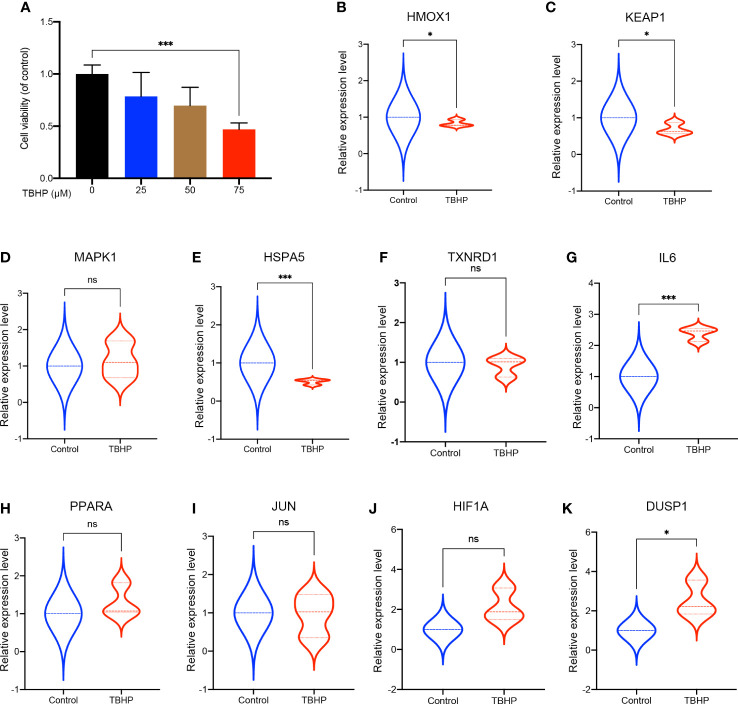
Validation of these hub FRDEGs in IDD cell model. **(A)** The cell viability of the rat NP cells treated with different concentrations of TBHP for 24 h was determined by the CCK-8 analysis. **(B–K)** The mRNA expression levels of these ten hub FRDEGs in the rat NP cells with or without TBHP treatment were determined by RT-qPCR assay. ns, not significant. ^∗^p < 0.05, ^∗∗∗^p < 0.001, n = 3.

To further validate the expression of theses hub FRDEGs in IDD, we established the IDD cell model by treating rat disc NP cells with TBHP. As shown in **Figure 7A**, the TBHP treatment for 24 h obviously inhibited the cell viability of rat NP cells in a dose-dependent manner, and the concentration of 75 μM was selected for subsequent experiments. Then we performed validation experiments for theses hub FRDEGs in the IDD cell model by RT-qPCR assay. As shown in **Figures 7B–K**, TBHP had significant effects on the mRNA expression levels of the hub FRDEGs. Compared to control group, the expression levels of HMOX1, KEAP1 and HSPA5 were downregulated in TBHP group, and the expression levels of IL6 and DUSP1 were upregulated in TBHP group.

## 4 Discussion

IDD is the one of the leading causes of LBP, which is a very common musculoskeletal disease and has brought a heavy healthcare burden and great socioeconomic cost globally (1–3). Ferroptosis is a newly discovered type of programmed cell death which is distinct from other types of cell death. More and more studies have demonstrated that ferroptosis is closely associated with multiple human diseases, especially degenerative skeletal diseases, including osteoarthritis, osteoporosis, and inflammatory arthritis (15). Interestingly, ferroptosis might also be interrelated with the pathogenesis and progression of IDD. In the present research, we performed comprehensive bioinformatic analysis to uncover the significant roles of ferroptosis in IDD. Firstly, we screened out the DGEs in IDD according to the public datasets, including 847 downregulated genes and 1422 upregulated genes. Then we found that the expression of ferroptosis-related DGEs in IDD group was different from that in control group. We have identified 80 FRDEGs, including 20 downregulated FRDEGs and 60 upregulated FRDEGs.

In this research work, we conducted functional enrichment analysis to investigate the enriched GO items and significant pathways based on these FRDEGs. These FRDEGs are mainly related to response to chemical, response to stress, and cellular response to chemical stimulus, *etc.* It is worth noting that the FRDEGs are closely associated with several important pathways in IDD, including TNF signaling pathway, HIF-1 signaling pathway, and IL-17 signaling pathway. TNF-α, a member of the tumor necrosis factor (TNF) superfamily, was reported to be dysregulated in degenerated IVDs (27). Moreover, TNF signaling is deeply involved in various pathological process during IDD, including extracellular matrix (ECM) degradation, apoptosis, autophagy inflammatory responses (27). Hypoxia-inducible factors (HIFs) are transcription factors that that plays essential roles in the cellular response to low oxygen (28). The hypoxia inducible factor 1 subunit alpha (*HIF1A*), one of the key FRDEGs identified in this study, was reported to be decreased with the disc degeneration and participated in the IDD process through interacting with autophagy (29). However, another research work by Wang et al. (30) demonstrated that HIF1A expression was dysregulated in cartilaginous endplate and annulus fibrosus tissues of IDD patients and mouse models. They also found that aberrant activation of HIF1A in EP and AF tissues was a pathological factor for DDD, and inhibition of its aberrant activation prevented the IDD development in animal models. Interleukin-17 (IL-17), namely IL-17A, is a key cytokine of IL-17 family and is primarily secreted by T helper 17 (Th17) cells. IL-17 can trigger various signal pathways to exert regulating effects on mammalian cells (31, 32). Importantly, the expression of IL-17 was positively correlated the degree of IDD, and it could promote the IDD progression by regulating ECM metabolism, inflammatory responses, neo-angiogenesis, and NP cell autophagy and proliferation (33). It is suggested that the FRDEGs might play roles in regulating these critical signaling pathways during IDD initiation and progression, which needs further investigations in the future.

Moreover, we conducted the PPI network analysis to further identify the key and hub genes among these FRDEGs. We determined ten most important hub FRDEGs, as follows: *HMOX1, KEAP1, MAPK1, HSPA5, TXNRD1, IL6, PPARA, JUN, HIF1A*, and *DUSP1*. Then we performed functional and pathway enrichment analysis to further explore the potential molecular functions of the ten hub genes. Importantly, these hub FRDEGs are primarily involved in response to oxidative stress and cellular response to oxidative stress. It is suggested that these hub FRDEGs are closely related to oxidative stress. Oxidative stress has played key roles in the pathogenesis and development of IDD, as reported in previous studies from ours and others (34–37). Interestingly, oxidative stress is closely associated with ferroptosis. In 2009, Reardon et al. (38) reported that iron injections could upregulate skeletal muscle iron content and promote oxidative stress in mice. Recently, researchers have found that iron overload was an independent risk factor for IDD and it accelerated IDD development *via* oxidative stress and ferroptosis in endplate chondrocytes (22). Besides, oxidative stress could induce ferroptosis in AF cells and NP cells in an autophagy‐dependent way during IDD process (39). Therefore, the interaction between oxidative stress and ferroptosis has significantly contributed to the disc degeneration. In the pathway enrichment analysis, the most meaningful and significantly enriched pathways include Th17 cell differentiation and HIF-1 signaling pathway, and related contents have been discussed above.

We further performed ROC analysis to evaluate the predictive value of the hub FRDEGs, and also established IDD cell model to validate these genes expression in the current study. Results of ROC analysis showed that six out of the ten hub FRDEGs might be potential signature genes for IDD, including *KEAP1, MAPK1, HSPA5, TXNRD1, JUN*, and *HIF1A*. KEAP1, the Kelch like ECH associated protein 1, has been found to exert promoting functions on ferroptosis (40). KEAP1 is a redox sensor for ROS and electrophiles and negatively modulates the activation of nuclear factor E2-related factor 2 (NRF2) signaling (41). In fact, the KEAP1-NRF2 complex is a redox-sensitive transcriptional regulatory system to protect cells against oxidative stress injury (42). In our previous review article, we have comprehensively discussed the roles of KEAP1-NRF2 system in IDD progression (4). This crucial antioxidant defense system could regulate the NP cell apoptosis, senescence, extracellular matrix (ECM) metabolism, inflammatory responses, and EP calcification in IDD. MAPK1, mitogen-activated protein kinase 1, is a core signal transductor of the MAPK/ERK signaling pathway, and it could facilitate ferroptosis through regulating ROS production (43). Evidence has shown that the MAPK1 expression was increased in the degenerated disc tissues and it could accelerate the development of disc degeneration (44). HSPA5, heat shock protein family A (Hsp70) member 5, was found to resist ferroptosis in cancer cells (45). Mechanistically, HSPA5 can bind to GPX4 protein and suppress the degradation of GPX4, which is a vital suppressor of intracellular lipid peroxidation and ferroptosis as described above. However, whether and how HSPA5 participates in IDD process is unclear so far. Though the effects of TXNRD1 (thioredoxin reductase 1) on ferroptosis is not fully understood, accumulating evidence has shown that TXNRD1 might also take part in the lipid peroxidation and ferroptosis (46, 47). The roles of TXNRD1 in IDD is not so clear either. JUN, namely Jun proto-oncogene, was demonstrated to inhibit erastin-induced ferroptosis in Schwann cells (48). A previous work has revealed that the c-Jun could alleviate IDD by modulating TGF-β (49). Previous studies have reported that HIF1A could exert promoting or inhibiting effects in regulating ferroptosis (50, 51). Moreover, as mentioned above, HIF1A has exerted critical regulatory functions in the disc degeneration process, and it could be also an important target to prevent the IDD development (29, 30, 52). Therefore, these key FRDEGs might be closely involved in the initiation and progression of IDD.

Accumulating studies have attempted to explore the underlying mechanisms of IDD by bioinformatic analysis, including inflammatory response, immune infiltration, mitochondrial dysfunction, etc (53, 54). The present study focused on the association between ferroptosis with IDD, and provided novel insights into the pathogenesis of IDD. However, certain limitations should be noted when interpreting our findings. First, we did not perform microarrays or RNA sequencing in this study. The gene expression data was obtained from the GEO database, and especially, the validation dataset was from blood samples of IDD patients and healthy controls. Second, the IDD cell model used in our study could not reflect the intact IDD microenvironment *in vivo*, which was far more complexed than *in vitro* models. And this may be part of the cause for some hub FRDEGs with different expression patterns in the microarray analysis and in the IDD cell model results. Lastly, the cellular and molecular biology experiments did not unveil specific mechanisms of ferroptosis in IDD, which needed more in-depth research to explore in the future.

## 5 Conclusion

In summary, this study illuminated that the expression pattern of ferroptosis related genes in IDD was distinct from controls. We have determined 80 FRDEGs dysregulated in IDD. Furthermore, ten most important OSRDEGs were identified and validated, including *HMOX1, KEAP1, MAPK1, HSPA5, TXNRD1, IL6, PPARA, JUN, HIF1A, DUSP1*. These hub FRDEGs had close relationships with oxidative stress and some other crucial signaling pathway, which were deeply involved in IDD initiation and development. These identified hub FRDEGs might be potential signature genes for IDD. This work reveals that ferroptosis might provide a novel and promising strategy for the diagnosis and treatment of the disc degeneration.

## Data availability statement

The original contributions presented in the study are included in the article/supplementary material. Further inquiries can be directed to the corresponding author.

## Author contributions

WL designed and supervised the study. QX and YZ performed the experiments, analyzed the data, and wrote the manuscript. All authors contributed to the article and approved the submitted version.

## References

[1] Maher C Underwood M Buchbinder R. Non-specific low back pain. Lancet (2017) 389(10070):736–47. doi: 10.1016/s0140-6736(16)30970-9 27745712

[2] Zhou Z Hui ES Kranz GS Chang JR de Luca K Pinto SM. Potential mechanisms underlying the accelerated cognitive decline in people with chronic low back pain: A scoping review. Ageing Res Rev (2022), 82:101767. doi: 10.1016/j.arr.2022.101767 36280211

[3] Binch ALA Fitzgerald JC Growney EA Barry F. Cell-based strategies for ivd repair: Clinical progress and translational obstacles. Nat Rev Rheumatol (2021) 17(3):158–75. doi: 10.1038/s41584-020-00568-w 33526926

[4] Xiang Q Zhao Y Lin J Jiang S Li W. The Nrf2 antioxidant defense system in intervertebral disc degeneration: Molecular insights. Exp Mol Med (2022) 54(8):1067–75. doi: 10.1038/s12276-022-00829-6 PMC9440120 35978054

[5] Sakai D Grad S. Advancing the cellular and molecular therapy for intervertebral disc disease. Adv Drug Delivery Rev (2015) 84:159–71. doi: 10.1016/j.addr.2014.06.009 24993611

[6] Sampara P Banala RR Vemuri SK Av GR Gpv S. Understanding the molecular biology of intervertebral disc degeneration and potential gene therapy strategies for regeneration: A review. Gene Ther (2018) 25(2):67–82. doi: 10.1038/s41434-018-0004-0 29567950

[7] Dixon SJ Lemberg KM Lamprecht MR Skouta R Zaitsev EM Gleason CE. Ferroptosis: An iron-dependent form of nonapoptotic cell death. Cell (2012) 149(5):1060–72. doi: 10.1016/j.cell.2012.03.042 PMC3367386 22632970

[8] Stockwell BR Friedmann Angeli JP Bayir H Bush AI Conrad M Dixon SJ. Ferroptosis: A regulated cell death nexus linking metabolism, redox biology, and disease. Cell (2017) 171(2):273–85. doi: 10.1016/j.cell.2017.09.021 PMC5685180 28985560

[9] Dixon SJ Stockwell BR. The role of iron and reactive oxygen species in cell death. Nat Chem Biol (2014) 10(1):9–17. doi: 10.1038/nchembio.1416 24346035

[10] Wu S Zhu C Tang D Dou QP Shen J Chen X. The role of ferroptosis in lung cancer. biomark Res (2021) 9(1):82. doi: 10.1186/s40364-021-00338-0 34742351 PMC8572460

[11] Yang WS SriRamaratnam R Welsch ME Shimada K Skouta R Viswanathan VS. Regulation of ferroptotic cancer cell death by Gpx4. Cell (2014) 156(1-2):317–31. doi: 10.1016/j.cell.2013.12.010 PMC4076414 24439385

[12] Ge C Zhang S Mu H Zheng S Tan Z Huang X. Emerging mechanisms and disease implications of ferroptosis: Potential applications of natural products. Front Cell Dev Biol (2021) 9:774957. doi: 10.3389/fcell.2021.774957 35118067 PMC8804219

[13] Toyokuni S Ito F Yamashita K Okazaki Y Akatsuka S. Iron and thiol redox signaling in cancer: An exquisite balance to escape ferroptosis. Free Radic Biol Med (2017) 108:610–26. doi: 10.1016/j.freeradbiomed.2017.04.024 28433662

[14] Conrad M Angeli JP Vandenabeele P Stockwell BR. Regulated necrosis: Disease relevance and therapeutic opportunities. Nat Rev Drug Discovery (2016) 15(5):348–66. doi: 10.1038/nrd.2015.6 PMC6531857 26775689

[15] Liu X Wang T Wang W Liang X Mu Y Xu Y. Emerging potential therapeutic targets of ferroptosis in skeletal diseases. Oxid Med Cell Longev (2022) 2022:3112388. doi: 10.1155/2022/3112388 35941905 PMC9356861

[16] Miao Y Chen Y Xue F Liu K Zhu B Gao J. Contribution of ferroptosis and Gpx4’s dual functions to osteoarthritis progression. EBioMedicine (2022) 76:103847. doi: 10.1016/j.ebiom.2022.103847 35101656 PMC8822178

[17] Lv Z Han J Li J Guo H Fei Y Sun Z. Single cell rna-seq analysis identifies ferroptotic chondrocyte cluster and reveals Trpv1 as an anti-ferroptotic target in osteoarthritis. EBioMedicine (2022) 84:104258. doi: 10.1016/j.ebiom.2022.104258 36137413 PMC9494174

[18] Hu Y Han J Ding S Liu S Wang H. Identification of ferroptosis-associated biomarkers for the potential diagnosis and treatment of postmenopausal osteoporosis. Front Endocrinol (Lausanne) (2022) 13:986384. doi: 10.3389/fendo.2022.986384 36105394 PMC9464919

[19] DeRuisseau KC Park YM DeRuisseau LR Cowley PM Fazen CH Doyle RP. Aging-related changes in the iron status of skeletal muscle. Exp Gerontol (2013) 48(11):1294–302. doi: 10.1016/j.exger.2013.08.011 PMC3812819 23994517

[20] Kim BJ Ahn SH Bae SJ Kim EH Lee SH Kim HK. Iron overload accelerates bone loss in healthy postmenopausal women and middle-aged men: A 3-year retrospective longitudinal study. J Bone Miner Res (2012) 27(11):2279–90. doi: 10.1002/jbmr.1692 22729843

[21] Zhang Y Han S Kong M Tu Q Zhang L Ma X. Single-cell rna-seq analysis identifies unique chondrocyte subsets and reveals involvement of ferroptosis in human intervertebral disc degeneration. Osteoarthr Cartilage (2021) 29(9):1324–34. doi: 10.1016/j.joca.2021.06.010 34242803

[22] Wang W Jing X Du T Ren J Liu X Chen F. Iron overload promotes intervertebral disc degeneration *Via* inducing oxidative stress and ferroptosis in endplate chondrocytes. Free Radical Biol Med (2022) 190:234–46. doi: 10.1016/j.freeradbiomed.2022.08.018 35981695

[23] Wan ZY Song F Sun Z Chen YF Zhang WL Samartzis D. Aberrantly expressed long noncoding rnas in human intervertebral disc degeneration: A microarray related study. Arthritis Res Ther (2014) 16(5):465. doi: 10.1186/s13075-014-0465-5 25280944 PMC4201740

[24] Wang Y Dai G Li L Liu L Jiang L Li S. Transcriptome signatures reveal candidate key genes in the whole blood of patients with lumbar disc prolapse. Exp Ther Med (2019) 18(6):4591–602. doi: 10.3892/etm.2019.8137 PMC6862187 31777557

[25] Li Y Pan D Wang X Huo Z Wu X Li J. Silencing Atf3 might delay tbhp-induced intervertebral disc degeneration by repressing npc ferroptosis, apoptosis, and ecm degradation. Oxid Med Cell Longev (2022) 2022:4235126. doi: 10.1155/2022/4235126 35480873 PMC9036167

[26] Kang L Xiang Q Zhan S Song Y Wang K Zhao K. Restoration of autophagic flux rescues oxidative damage and mitochondrial dysfunction to protect against intervertebral disc degeneration. Oxid Med Cell Longev (2019) 2019:7810320. doi: 10.1155/2019/7810320 31976028 PMC6954474

[27] Wang C Yu X Yan Y Yang W Zhang S Xiang Y. Tumor necrosis factor-A: A key contributor to intervertebral disc degeneration. Acta Biochim Biophys Sin (2017) 49(1):1–13. doi: 10.1093/abbs/gmw112 27864283

[28] McGettrick AF O’Neill LAJ. The role of hif in immunity and inflammation. Cell Metab (2020) 32(4):524–36. doi: 10.1016/j.cmet.2020.08.002 32853548

[29] He R Wang Z Cui M Liu S Wu W Chen M. Hif1a alleviates compression-induced apoptosis of nucleus pulposus derived stem cells *Via* upregulating autophagy. Autophagy (2021) 17(11):3338–60. doi: 10.1080/15548627.2021.1872227 PMC8632345 33455530

[30] Wang Z Chen H Tan Q Huang J Zhou S Luo F. Inhibition of aberrant Hif1α activation delays intervertebral disc degeneration in adult mice. Bone Res (2022) 10(1):2. doi: 10.1038/s41413-021-00165-x 34983922 PMC8727577

[31] Konieczny P Xing Y Sidhu I Subudhi I Mansfield KP Hsieh B. Interleukin-17 governs hypoxic adaptation of injured epithelium. Science (2022) 377(6602):eabg9302. doi: 10.1126/science.abg9302 35709248 PMC9753231

[32] Mimpen JY Baldwin MJ Cribbs AP Philpott M Carr AJ Dakin SG. Interleukin-17a causes osteoarthritis-like transcriptional changes in human osteoarthritis-derived chondrocytes and synovial fibroblasts in vitro. Front Immunol (2021) 12:676173. doi: 10.3389/fimmu.2021.676173 34054865 PMC8153485

[33] Tan JH Li ZP Liu LL Liu H Xue JB. Il-17 in intervertebral disc degeneration: Mechanistic insights and therapeutic implications. Cell Biol Int (2022) 46(4):535–47. doi: 10.1002/cbin.11767 35066966

[34] Zhao Y Xiang Q Lin J Jiang S Li W. Oxidative stress in intervertebral disc degeneration: New insights from bioinformatic strategies. Oxid Med Cell Longev (2022) 2022:2239770. doi: 10.1155/2022/2239770 35401932 PMC8991415

[35] Song Y Li S Geng W Luo R Liu W Tu J. Sirtuin 3-dependent mitochondrial redox homeostasis protects against ages-induced intervertebral disc degeneration. Redox Biol (2018) 19:339–53. doi: 10.1016/j.redox.2018.09.006 PMC6139007 30216853

[36] Li Y Chen L Gao Y Zou X Wei F. Oxidative stress and intervertebral disc degeneration: Pathophysiology, signaling pathway, and therapy. Oxid Med Cell Longev (2022) 2022:1984742. doi: 10.1155/2022/1984742 36262281 PMC9576411

[37] Kang L Liu S Li J Tian Y Xue Y Liu X. The mitochondria-targeted anti-oxidant mitoq protects against intervertebral disc degeneration by ameliorating mitochondrial dysfunction and redox imbalance. Cell Prolif (2020) 53(3):e12779. doi: 10.1111/cpr.12779 32020711 PMC7106957

[38] Reardon TF Allen DG. Iron injections in mice increase skeletal muscle iron content, induce oxidative stress and reduce exercise performance. Exp Physiol (2009) 94(6):720–30. doi: 10.1113/expphysiol.2008.046045 19201785

[39] Yang RZ Xu WN Zheng HL Zheng XF Li B Jiang LS. Involvement of oxidative stress-induced annulus fibrosus cell and nucleus pulposus cell ferroptosis in intervertebral disc degeneration pathogenesis. J Cell Physiol (2021) 236(4):2725–39. doi: 10.1002/jcp.30039 PMC7891651 32892384

[40] Fan Z Wirth AK Chen D Wruck CJ Rauh M Buchfelder M. Nrf2-Keap1 pathway promotes cell proliferation and diminishes ferroptosis. Oncogenesis (2017) 6(8):e371. doi: 10.1038/oncsis.2017.65 28805788 PMC5608917

[41] Itoh K Wakabayashi N Katoh Y Ishii T Igarashi K Engel JD. Keap1 represses nuclear activation of antioxidant responsive elements by Nrf2 through binding to the amino-terminal Neh2 domain. Genes Dev (1999) 13(1):76–86. doi: 10.1101/gad.13.1.76 9887101 PMC316370

[42] Yamamoto M Kensler TW Motohashi H. The Keap1-Nrf2 system: A thiol-based sensor-effector apparatus for maintaining redox homeostasis. Physiol Rev (2018) 98(3):1169–203. doi: 10.1152/physrev.00023.2017 PMC9762786 29717933

[43] Su L Jiang X Yang C Zhang J Chen B Li Y. Pannexin 1 mediates ferroptosis that contributes to renal Ischemia/Reperfusion injury. J Biol Chem (2019) 294(50):19395–404. doi: 10.1074/jbc.RA119.010949 PMC6916502 31694915

[44] Zhou M He SJ Liu W Yang MJ Hou ZY Meng Q. Ezh2 upregulates the expression of Mapk1 to promote intervertebral disc degeneration *Via* suppression of mir-129-5p. J Gene Med (2022) 24(3):e3395. doi: 10.1002/jgm.3395 34668273

[45] Zhu S Zhang Q Sun X Zeh HJ 3rd Lotze MT Kang R. Hspa5 regulates ferroptotic cell death in cancer cells. Cancer Res (2017) 77(8):2064–77. doi: 10.1158/0008-5472.Can-16-1979 PMC5392369 28130223

[46] Tang D Chen X Kang R Kroemer G. Ferroptosis: Molecular mechanisms and health implications. Cell Res (2021) 31(2):107–25. doi: 10.1038/s41422-020-00441-1 PMC8026611 33268902

[47] Guo W Wu Z Chen J Guo S You W Wang S. Nanoparticle delivery of mir-21-3p sensitizes melanoma to anti-Pd-1 immunotherapy by promoting ferroptosis. J Immunother Cancer (2022) 10(6). doi: 10.1136/jitc-2021-004381 PMC9226924 35738798

[48] Gao D Huang Y Sun X Yang J Chen J He J. Overexpression of c-jun inhibits erastin-induced ferroptosis in schwann cells and promotes repair of facial nerve function. J Cell Mol Med (2022) 26(8):2191–204. doi: 10.1111/jcmm.17241 PMC8995448 35191156

[49] Lei M Wang K Li S Zhao K Hua W Wu X. The c-jun signaling pathway has a protective effect on nucleus pulposus cells in patients with intervertebral disc degeneration. Exp Ther Med (2020) 20(5):123. doi: 10.3892/etm.2020.9251 33005249 PMC7523272

[50] Yang YC Zhang MY Liu JY Jiang YY Ji XL Qu YQ. Identification of ferroptosis-related hub genes and their association with immune infiltration in chronic obstructive pulmonary disease by bioinformatics analysis. Int J Chron Obstruct Pulmon Dis (2022) 17:1219–36. doi: 10.2147/copd.S348569 PMC9148178 35637927

[51] Yang M Chen P Liu J Zhu S Kroemer G Klionsky DJ. Clockophagy is a novel selective autophagy process favoring ferroptosis. Sci Adv (2019) 5(7):eaaw2238. doi: 10.1126/sciadv.aaw2238 31355331 PMC6656546

[52] Li Y Liu S Pan D Xu B Xing X Zhou H. The potential role and trend of Hif−1α in intervertebral disc degeneration: Friend or foe? (Review). Mol Med Rep (2021) 23(4). doi: 10.3892/mmr.2021.11878 PMC7893690 33537810

[53] Lan T Hu Z Guo W Yan B Zhang Y. Development of a novel inflammatory-associated gene signature and immune infiltration patterns in intervertebral disc degeneration. Oxid Med Cell Longev (2022) 2022:2481071. doi: 10.1155/2022/2481071 36193061 PMC9526649

[54] Zhu Z He Z Tang T Wang F Chen H Li B. Integrative bioinformatics analysis revealed mitochondrial dysfunction-related genes underlying intervertebral disc degeneration. Oxid Med Cell Longev (2022) 2022:1372483. doi: 10.1155/2022/1372483 36267810 PMC9578809

